# Effects of Different Ranges of Loads on Physical Performance Using Velocity-Based Resistance Training

**DOI:** 10.3390/sports13040121

**Published:** 2025-04-16

**Authors:** Javier Riscart-López, Juan Sánchez-Valdepeñas, Fernando Colomina-Clemens, Esteban Crespo-García, Guillermo de Castro-Maqueda, Miguel Ángel Rosety-Rodríguez, Juan Antonio León-Prados, Fernando Pareja-Blanco

**Affiliations:** 1 Department of Sports and Computer Sciences, Faculty of Sport Sciences, Universidad Pablo de Olavide, 41013 Seville, Spain; javiriscart@gmail.com (J.R.-L.); jaleopra@upo.es (J.A.L.-P.); fparbla@upo.es (F.P.-B.); 2 Department of Physical Education, Faculty of Education Sciences, University of Cádiz, 11519 Puerto Real, Spain; guillermoramon.decastro@uca.es; 3 Science-Based Training Research Group, Physical Performance & Sports Research Center (CIRFD), Universidad Pablo de Olavide, 41013 Seville, Spain; juan_valdemate@hotmail.com (J.S.-V.); colomina.jijona@gmail.com (F.C.-C.); ecg806@hotmail.com (E.C.-G.)

**Keywords:** strength training, dose–response, athletic performance, training programming, load monitoring, training prescription

## Abstract

(1) Background: The range of loads is defined as the difference between the highest and the lowest relative load (i.e., %1RM) used throughout a resistance training program. However, the optimal range of loads has not been studied yet. Thus, the aim of this study was to compare the effects of different ranges of load (from 50 to 85% 1RM (R_50–85_), from 55 to 75% 1RM (R_55–75_), and from 60 to 70% 1RM (R_60–70_) on physical performance using velocity-based resistance training (VBT). (2) Methods: Thirty-eight men (mean ± standard deviation; age: 23.3 ± 3.6 years, body mass: 76.5 ± 8.3 kg, and height: 1.77 ± 0.04 m) were randomly assigned to R_50–85_, R_55–75_ and R_60–70_ groups and followed an 8-week VBT intervention using the full squat (SQ) exercise. All groups trained with similar mean relative intensity (65% 1RM) and total volume (240 repetitions). Pre- and post-training measurements included the following: in the SQ exercise, 1RM load, the average velocity attained for all absolute loads common to pre-tests and post-tests (AV), and the average velocity for those loads that were moved faster (AV > 1) and slower (AV < 1) than 1 m·s^−1^ at Pre-training tests. Moreover, countermovement jump (CMJ) height and 10 m (T10), 20 m (T20), and 10–20 m (T10–20) running sprint times were measured. (3) Results: Significant group x time interactions were observed in AV (*p* ≤ 0.01), where R_50–85_ obtained significantly greater gains than R_60–70_ (*p* ≤ 0.05). All groups attained significant increases in 1RM, AV, AV > 1, AV < 1, and CMJ (*p* ≤ 0.001–0.005). Significant improvements were observed in running sprint for R_60–70_ in T10–20 and R_60–70_ in T20 and T10–20 (*p* ≤ 0.05), but not for R_50–85_. (4) Conclusions: Different ranges of loads induce distinct strength adaptions. Greater ranges of loads resulted in greater strength gains in the entire load-velocity spectrum. However, in high-velocity actions, such as sprinting, significant enhancements were observed only for smaller ranges of loads. Coaches and strength and conditioning professionals could use a range of loads according to the time-related criterion (i.e., proximity or number of future competitions), enabling better adaptation and increasing physical performance at a specific time.

## 1. Introduction

A well-designed and implemented resistance training (RT) program increases muscular strength, potentially boosting athletic performance [1,2,3,4]. The suitable manipulation of RT variables, such as exercise type, intensity, sets and repetitions, rest duration, and movement velocity, is essential for prescribing an effective RT program [5,6,7]. Among the RT variables, one variable that has not yet been explored is the range of loads used in an RT program. The range of loads refers to the difference between the highest and lowest percentages of one-repetition maximum (%1RM) used throughout an RT program.

The previous literature has shown that training with heavy-load training yields greater benefits on 1RM than training with lighter loads [8,9,10]. In contrast, lighter loads provide superior gains in sprint performance than heavy-load training [11,12]. Accordingly, while different studies have analyzed the effects of various loading magnitudes [8,9,10,11], the optimal load range for an RT program remains unknown.

To accurately compare different ranges of loads, the average loading magnitude achieved during the training program (i.e., mean relative intensity, MRI) should be matched among training programs [13]. The MRI represents the weighted average repetitions performed at each %1RM [13]. However, this metric does not consider the level of effort exerted within each set (i.e., proximity to task failure) [14], meaning that the same MRI can be attained through different set configurations, potentially leading to distinct long-term adaptations.

Velocity-based training (VBT) is an RT approach that uses movement velocity as a key metric to regulate load, intensity, and fatigue [14,15,16]. Unlike traditional percentage-based methods, which rely on predetermined loads relative to an athlete’s 1RM, VBT provides real-time feedback on bar speed, allowing for individualized adjustments based on daily performance fluctuations [15]. Therefore, VBT enables researchers and coaches to align the actual load used during training sessions with the scheduled load. This makes VBT particularly useful for matching MRI across training programs to investigate the effects of the load range on training adaptations.

In light of this information, the aim of this study was to compare the effects of various load ranges: from 50 to 85% 1RM (35%: R_50–85_), from 55 to 75% 1RM (20%: R_55–75_), and from 60 to 70% 1RM (10%: R_60–70_) on physical performance using a VBT approach. Based on the previous literature, it was hypothesized that broader ranges of loads would lead to superior gains in performance for slow-velocity actions (i.e., 1RM), while smaller load ranges would induce greater improvements in high-velocity actions (i.e., running sprint).

## 2. Materials and Methods

### 2.1. The Experimental Approach to the Problem

An experimental longitudinal research design was carried out to explore the effects of three ranges of loads: (1) from 50 to 85% 1RM (35%: R_50–85_), (2) from 55 to 75% 1RM (20%: R_55–75_), and (3) from 60 to 70% 1RM (10%: R_60–70_), on physical performance. Participants trained twice a week (at least 48 h apart), following a linear VBT programming model based on the SQ exercise for 8 weeks. Physical performance was assessed on two distinct occasions: the week before (pre-training) and after (post-training) the VBT intervention. The following tests were performed: sprint, vertical countermovement jump (CMJ), and progressive loading SQ test. The researcher conducting the assessments was blinded to the participants’ group allocating. Participants were required not to engage or participate in any other type of strenuous physical activity or competitive events throughout the investigation. Participants were motivated to give maximal effort with strong verbal encouragement during all testing and training sessions. Sessions were conducted in a research laboratory under the supervision of experienced investigators, at the same time of day (±1 h) and with similar environmental conditions (~20 °C and ~60% humidity).

### 2.2. Participants

Thirty-eight men volunteered to participate in this study. All participants were physically active sports science students with RT experience (isometric, isotonic, isokinetic, and bodyweight training), ranging from 1.5 to 4 years, and apt to perform the SQ with proper technique. Subjects were highly familiarized with the VBT approach, and they had previously participated in studies and training sessions in our facilities, supervised by our research group. Participants were randomly assigned to one of three experimental groups, based on their baseline 1RM. Throughout the study, four participants dropped out due to injury or illness (not related to the training intervention), so the remaining participants per group were R_50–85_ (*n* = 12), R_55–75_ (*n* = 12), and R_60–70_ (*n* = 10). The participants’ characteristics are presented in Table 1. Once informed about the purpose, procedures, and potential risks of the investigation, all participants gave their voluntary written consent to participate. The participants had no physical limitations, health problems, or musculoskeletal injuries that could affect the evaluation. The present study was approved by the Research Ethics Committee of “Hospitales Universitarios Virgen Macarena-Virgen del Rocío” (Reference: 1547-N-18) in accordance with the Declaration of Helsinki.

### 2.3. Testing Procedures

Anthropometric measurements were determined using a weight scale and stadiometer (for body mass and height) and were taken prior to the physical testing. Physical performance was evaluated at pre- and post-training using a battery of tests performed in a single session in a fixed sequence, as described below. Pre-training assessments were conducted after 24 h of rest, while post-training assessments were conducted after 4 days following the last training session.

#### 2.3.1. Sprint Test

Two 20 m maximal sprints, separated by 3 min of rest, were performed on an indoor running track. Photocell timing gates (Witty, Microgate, Bolzano, Italy) were placed at 0, 10, and 20 m so that the times to cover 0–10 m (T10), 0–20 m (T20), and 10–20 m (T10–20) could be determined. Participants started from a standing position, 1 m before the starting line. A standardized warm-up protocol was conducted, which incorporated several sets of progressively faster 20 m running accelerations. The best time in each split time was used for further analysis. Test-retest reliability was as follows: coefficient of variation (CV) of 1.7%, 0.7%, and 2.4%, for T10, T20, and T10–20, respectively. The intraclass correlation coefficient (ICC) with a 95% confidence interval (CI) was 0.964 (0.928–0.982) for T10, 0.988 (0.976–0.994) for T20, and 0.932 (0.861–0.966).

#### 2.3.2. Countermovement Jump Test

Jump height was determined using an infrared timing system (OptojumpNext, Microgate, Bolzano, Italy). Subjects were instructed to jump with both hands resting on the hip, perform countermovement between eccentric and concentric phases, and try to reach the maximum height with the correct technique. Subjects performed five maximal CMJs, interspersed with 45 s rests. The highest and lowest CMJ height values were discarded, and the mean value of the 3 remaining CMJs was used for analysis. The warm-up consisted of 2 sets of 10 squats without external loads and at controlled velocity, 5 submaximal CMJs, and 3 close-to-maximal CMJs. CV was 1.5%, and ICC was 0.996 (95% CI: 0.993–0.998).

#### 2.3.3. Progressive Loading Squat Test

A progressive loading test was conducted in the SQ exercise. A Smith-machine (Multipower Fitness Line, Peroga, Murcia, Spain) without a counterweight mechanism was used. Repetitions were recorded using a linear velocity transducer (T-Force System Ergotech, Murcia, Spain), which is valid and reliable [17]. The SQ was performed with participants starting from an upright position, with their knees and hips fully extended, feet parallel and about shoulder-width apart, and the barbell resting across the back at the acromion level. Each participant descended smoothly to their maximum depth (approximately 35–40° knee flexion) and immediately ascended as fast as possible. The warm-up included two sets of eight and six SQs, with 3 min of rest between sets, using loads of 20 kg and 30 kg, respectively. The initial load was set to 30 kg and was progressively increased by 10 kg until the measured mean propulsive velocity (MPV) fell below 0.5 m·s^−1^. The 1RM was estimated from the MPV with the heaviest load (kg) recorded during the tests, as follows: 100 × LOAD/−5.961 × MPV^2^ − 50.71 × MPV + 117 R^2^ = 0.954 (Standard Error of Estimate = 4.02%) [16]. Three repetitions were conducted for lighter loads (≤50% 1RM), two for moderate loads (50–70% 1RM), and just one for heavier loads (>80% 1RM). Participants received strong verbal encouragement to motivate them to exert maximal effort. Inter-set recoveries ranged from 3 (light loads) to 5 min (heavy loads). The fastest MPV achieved for each load was considered for further analysis. All reported velocities in this study correspond to the mean velocity of the propulsive phase of each repetition [17]. Moreover, MPVs were categorized into different portions of the load–velocity spectrum: (a) average MPV achieved against all absolute loads common to pre- and post-tests (AV); (b) average MPV achieved against absolute loads moving faster than 1 m·s^−1^ at pre-training (AV > 1, ‘light’ loads); and (c) average MPV achieved against absolute loads moving slower than 1 m·s^−1^ at pre-training (AV < 1, ‘heavy’ loads).

#### 2.3.4. Resistance Training Program

The three groups trained twice a week (48–72 h apart) for 8 weeks, performing exclusively the SQ exercise, with similar MRI (65% 1RM), total repetitions (240), sets (3), and inter-set recoveries (4 min). The main characteristics of the three VBT programs are presented in Table 2. The difference among the groups was the range of loads (R_50–85_: from 50 to 85% 1RM, R_55–75_: from 55 to 75% 1RM, R_60–70_: from 60 to 70% 1RM). Relative loads were determined from the load-velocity relationship for the SQ (16). Thus, a target MPV to be reached in the fastest repetition of each training session was used to estimate the scheduled %1RM. The absolute load (kg) was individually adjusted to match the velocity (±0.03 m·s^−1^) associated with the %1RM prescribed for each session. A standardized warm-up preceded all RT sessions as follows: 5 min of jogging, two sets of 10 SQs without additional load, and 6, 4, and 3 SQs with 40%, 50%, and 60% 1RM, respectively, for sessions where the scheduled relative load was equal to or lighter than 70% 1RM. Two SQ repetitions at 70% 1RM were added for sessions in which the relative intensity exceeded 70% 1RM, and one repetition at 80% 1RM was included for sessions where the relative intensity surpassed 80% 1RM. A 3 min rest interval was used between all sets. MRI was calculated as the average weight lifted under all loading conditions across all sets and repetitions. To compare the actual training program conducted for each range of loads, based on previous research [18], the following variables were calculated: Fastest-MPV, average of the fastest repetition measured in each session, which corresponds with the intensity (%1RM) scheduled; Average-MPV, average MPV attained during the entire training program (excluding warm-up); VL, velocity loss induced in the set; Total Reps, total number of repetitions performed during the training program (excluding warm-up); MRI, weighted average of the number of repetitions performed with each %1RM; NTF, number of times to failure; Rep per set with a given %1RM, average number of repetitions performed in each set with each relative load; VL per set with a given %1RM, VL attained in each set with each relative load. Furthermore, the evolution of the strength performance across training sessions was estimated from the load–velocity relationship [16], using the fastest repetition at 60% 1RM during the warm-up. This load was selected because it was used in all training sessions by all groups. Therefore, the potential error in the load estimation was similar for all groups.

### 2.4. Statistical Analyses

Values are reported as means ± standard deviation (SD). Statistical significance was established at the *p* ≤ 0.05 level. The normal distribution of the variables at pre-training was assessed using the Shapiro–Wilk test, while the homogeneity of variance among groups was verified using Levene’s test. An ICC with a 95% CI using the one-way random effects model was used to determine the between-participant reliability. Within-participant variation was determined by calculating the CV. Data were analyzed using a 3 × 2 factorial ANOVA with Bonferroni’s post hoc comparisons using one between-group factor (R_50–85_, R_55–75,_ and R_60–70_) and one within-group factor (pre- vs. post-training). In addition, data were tested using the Magnitude-Based Decisions approach [19]. Effect size (ES) was calculated using Hedge’s g [20]. Probabilities were also calculated to determine whether the true (unknown) differences were lower, similar, or higher than the smallest worthwhile difference or change (0.2 multiplied by the between-subject SD [21]). The remaining analyses were performed using the SPSS 18.0 Statistical Software Package (SPSS Inc., Chicago, IL, USA).

## 3. Results

**Figure 1:**
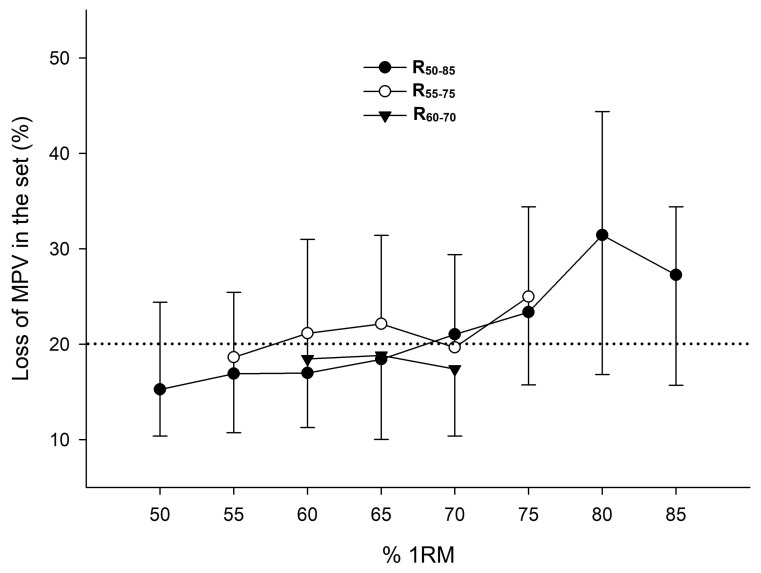
Velocity loss against different %1RM induced by each range of load. MPV: mean propulsive velocity. Bars indicate the standard deviation; *n* = 34. R_50–85_: range from 50 to 85% 1RM (*n* = 12). R_55–75_: range from 55 to 75% 1RM (*n* = 12). R_60–70_: range from 60 to 70% 1RM (*n* = 10).

Training compliance was 100% for all participants who completed the intervention. Descriptive characteristics of the three training interventions conducted are reported in Table 3. All groups were trained with the same MRI (~65% 1RM) and Average-MPV (~0.96 m·s^−1^). No significant differences between groups were observed in the average VL. R_60–70_ achieved a higher Fastest-MPV than R_55–75_ and R_50–85_ (*p* < 0.01), and R_55–75_ achieved a higher Fastest-MPV than R_50–85_ (*p* < 0.01). R_60–70_ accumulated more Total Reps and less NTF than R_50–85_ (*p* < 0.05). All groups performed the same Rep per set for every common %1RM. No significant differences between groups were observed in the VL experienced against the different %1RM (Figure 1).

### 3.1. Running Sprint and Vertical Jump

**Figure 2:**
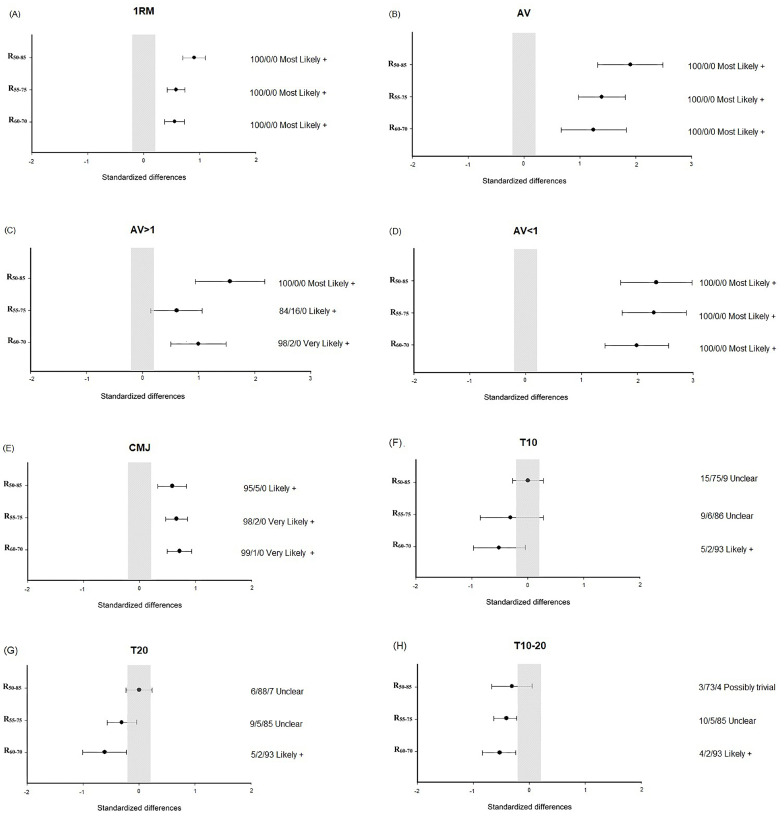
Intragroup changes in (**A**) 1RM: one-repetition maximum in the full-squat exercise. (**B**) AV: average mean propulsive velocity (MPV) attained against absolute loads common to pre- and post-test in the progressive loading squat test. (**C**) AV > 1: average MPV attained against absolute loads moved faster than 1 m·s^−1^ at pre-training. (**D**) AV < 1: average MPV attained against absolute loads moved slower than 1 m·s^−1^ at pre-training. (**E**) CMJ: countermovement jump height. (**F**) T10: 10 m running sprint time. (**G**) T20: 20 m running sprint time. (**H**) T10–20: 10–20 m running sprint time in R_50–85_: range from 50 to 85% 1RM (*n* = 12). R_55–75_: range from 55 to 75% 1RM (*n* = 12). R_60–70_: range from 60 to 70% 1RM (*n* = 10). Bars indicate uncertainty in true mean differences with 90% confidence intervals. The trivial area was calculated from the smallest worthwhile change.

No significant ‘group’ × ‘time’ interactions were observed for CMJ height and any sprint times. A significant time effect was noted for CMJ height and all sprint times (*p* < 0.05–0.001) (Table 4). All groups enhanced their CMJ height (*p* < 0.001). R_55–75_ and R_60–70_ improved T10–20, but no significant changes were observed for R_50–85_. Only R_60–70_ showed significant improvements in T20 (*p* < 0.01). No significant improvements were achieved in T10 for any groups. R_60–70_ obtained the greatest ES and percent changes in running sprint and CMJ (Figure 2 and Table 4).

### 3.2. Progressive Loading Squat Test

**Figure 3:**
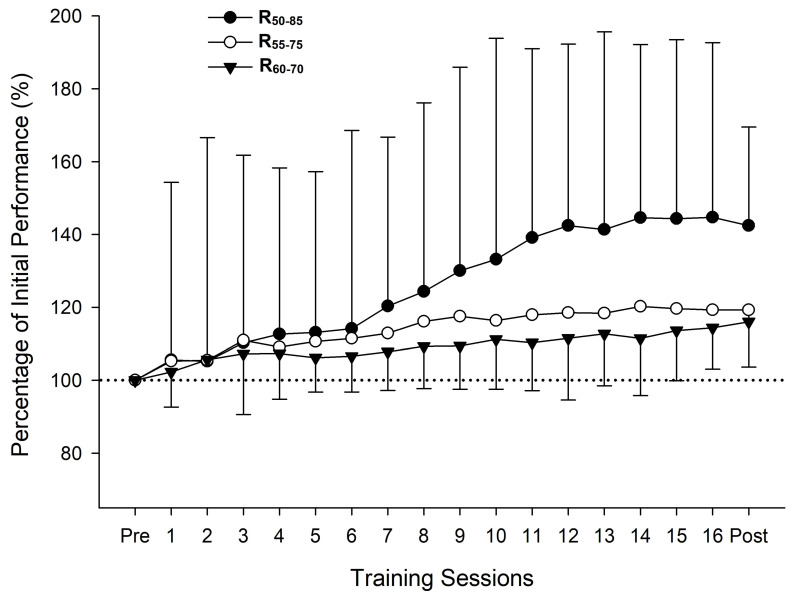
Evolution of performance in each training session of the three ranges of loads under study. Bars indicate the standard deviation; *n* = 34. R_50–85_: range from 50 to 85% 1RM (*n* = 12). R_55–75_: range from 55 to 75% 1RM (*n* = 12). R_60–70_: range from 60 to 70% 1RM (*n* = 10).

A significant time effect was found for all SQ variables analyzed (*p* < 0.001). A significant group x time interaction was observed for AV (*p* < 0.05), where R_50–85_ obtained significantly greater gains than R_60–70_ (*p* < 0.05). After training, all groups significantly improved 1RM, AV, AV > 1, and AV < 1 (*p* < 0.001–0.01) (Table 4). R_50–85_ obtained the greatest ES and percent changes in all SQ variables (Figure 2 and Table 4). The performance throughout the training program for the three training groups is reported in Figure 3.

## 4. Discussion

This is the first study to compare the effects of different ranges of load on physical performance. One of the most relevant aspects of the present study was the method used to match the efforts performed by the three experimental groups. The current study matched the MRI achieved at each load range and ensured that the different % contributing to this MRI were attained in every training session through a VBT approach. Lifting velocity was measured and recorded for every repetition throughout the intervention, allowing the strict and individualized control of training intensity in every training session. Overall, it was observed that different load ranges induce distinct strength adaptations. Therefore, our hypothesis that greater ranges of loads would induce superior performance gains in slow velocity actions (i.e., 1RM), while smaller ranges of loads would attain greater enhancements in high velocity actions (i.e., running sprint), was confirmed. In this regard, broader load ranges (R_50–85_ ≥ R_55–75_ ≥ R_60–70_) led to similar or better gains in performance in slow velocity actions (i.e., 1RM, AV, AV > 1, and AV < 1). However, in high-velocity actions (i.e., CMJ, T10, T20, and T10–20), similar or greater enhancements were noted for smaller ranges of loads (R_60–70_ ≥ R_55–75_ ≥ R_50–85_). This study design allows for isolating the independent variable (i.e., range of load) while matching the rest of the training variables (MRI [65% 1RM], level of effort [~20% VL, i.e., about half-maximal repetitions], volume [240 repetitions], rest time [4 min], among others). Therefore, it is reasonable to suggest that the different results obtained regarding muscle strength, jumping, and sprint performance gains were due to the distinct ranges of load used throughout the training program.

Regarding SQ performance, all groups exhibited significant gains in 1RM and other strength-related variables, reinforcing the effectiveness of RT across different loading strategies. Notably, the R_50–85_ group, which utilized the broadest range of loads, achieved the greatest improvements in strength-related parameters compared to the narrower load range groups. These results may be attributed to the training principles of specificity and overload. Although MRI was matched among groups (65% 1RM), the R_50–85_ group trained with heavier loads in some sessions (85% 1RM), which could have led to superior effects on 1RM strength. Additionally, the broader loading spectrum used by this group (from 50 to 85% of 1RM) may have stimulated adaptations across the distinct zones of the load-velocity relationship. Therefore, strength adaptations resulting from an RT program may be specific to the region of the load–velocity relationship being trained [8,9,10,22,23]. Additionally, these findings can be understood through the greater 1RM gains seen at larger ranges (R_50–85_ > R_55–75_ > R_60–70_). Since AV, AV > 1, and AV < 1 are computed using the same absolute weights during Pre-training and Post-training assessments, for participants who experienced higher increases in their 1RM, the same absolute load (kg) signifies a lighter relative load (%1RM) [24]. Therefore, it is reasonable to conclude that they can lift lighter relative loads at faster velocities.

In terms of athletic adaptations, smaller load ranges (R_60–70_ > R_55–75_ > R_50–85_) resulted in greater improvements in high-velocity activities (CMJ, T10, T20, and T10–20), achieving higher ESs and percentage changes (Figure 2 and Table 4). This may stem from the fatigue experienced during the training program, as R_50–85_ generated a higher volume load (21.4% vs. 18.1% for R_50–85_ and R_60–70_, respectively) (Table 3). As shown in Figure 1, the average fatigue caused by R_50–85_ at higher loads (>75% 1RM) surpassed the average from total training. Consequently, training close to muscle failure, particularly during the latter half of the program (as noted in Table 3 and Figure 1), can negatively impact the enhancement of high-velocity actions [25]. In contrast, R_55–75_ and R_60–70_ generally maintained a distance from exhaustion, with R_60–70_ demonstrating greater improvements than R_55–75_ (Table 4 and Figure 2). The superior gains seen with R_60–70_ over R_55–75_ may be attributed to the significantly faster mean propulsive velocity achieved with R_60–70_ (Table 3). Supporting our findings, earlier research has indicated comparable enhancements in high-velocity actions when utilizing faster training speeds as opposed to slower ones, potentially facilitating a better transfer of performance [11,26].

Remarkably, the relative load was prescribed on a daily basis by adjusting the absolute load (kg) according to the MPV associated with the %1RM prescribed for each training session [16]. This approach allowed the measurement of strength performance during each training session (Figure 3). On this point, no statistically significant differences were found between groups regarding the performance evolution during the intervention. However, R_50–85_ appears to induce greater improvements in SQ strength from the middle to the end of the training program. In line with these results, again, it seems that the specificity of the loads used (>70% 1RM) from the middle to the last training program (Table 2) accomplished by R_50–85_ induces higher strength adaptations in SQ compared to other groups [26,27].

From a methodological standpoint, VBT proved to be a valuable tool in maintaining consistency across training programs by regulating intensity in real time. The ability to align actual training loads with scheduled prescriptions ensured that observed differences in performance outcomes were attributable to load range variations rather than discrepancies in MRI. This highlights the practical relevance of VBT in RT research and its application in individualized strength and conditioning programs.

Regarding potential limitations, the study sample consisted of moderately resistance-trained men. Consequently, the changes observed among the three VBT training schemes could differ in other populations (i.e., top-level athletes or athletes with significantly different relative strength values). The findings can only be understood in relation to the analyzed exercise (SQ). In addition, a Smith machine was used for both testing and training. This could be a practical limitation of our design as most athletes in real-life training use free weights. No physiological indicators (electromyographic activity or structural changes) were measured, which have limited the interpretation and explanation of results obtained.

## 5. Conclusions

In conclusion, three different load ranges using a VBT approach applied over 8 weeks seem suitable for enhancing physical performance. However, increasing the load ranges using heavier %1RMs resulted in similar or even superior performance gains in slow velocity actions (1RM, AV, AV > 1, and AV < 1). Conversely, reducing the range of loads by avoiding heavy loads and their associated slow repetitions resulted in similar or even superior gains in performance in high-velocity actions (CMJ, T10, T20, and T10–20).

## 6. Practical Applications

The choice of the load range used is a variable to consider when designing an RT program. Depending on the specific training goal being pursued, a range of loads should be selected in advance. Therefore, coaches and strength and conditioning professionals may use smaller load ranges to maximize strength adaptations in high-velocity actions, such as jumping and sprinting. However, wider load ranges should be employed to enhance strength-related adaptations across different parts of the load-velocity relationship. Moreover, broader load ranges exhibit a more pronounced time-course of SQ strength increases compared to smaller load ranges. This aspect is significant when selecting a load range based on the time-related criterion (i.e., proximity or number of upcoming competitions), enabling better adaptation and improving physical performance at a specific time.

## Figures and Tables

**Table 1:** Descriptive characteristics of the participants belonging to each training group.

	R_50–85_ (*n* = 12)	R_55–75_ (*n* = 12)	R_60–70_ (*n* = 10)
Age (years)	23.8 ± 3.2	22.5 ± 2.8	22.2 ± 4.7
Height (m)	1.77 ± 0.04	1.78 ± 0.02	1.77 ± 0.05
Body mass (kg)	79.2 ± 7.7	76.5 ± 11.4	74.2 ± 7.7

Data are mean ± SD, *n* = 34. R_50–85_: load range from 50 to 85% 1RM (*n* = 12). R_55–75_: load range from 55 to 75% 1RM (*n* = 12). R_60–70_: load range from 60 to 70% 1RM (*n* = 10).

**Table 2:** Descriptive characteristics of the 8-week velocity-based squat training program scheduled for each range of loads.

Intensity Scheduled		Session 1	Session 2	Session 3	Session 4	Session 5	Session 6	Session 7	Session 8
R_50–85_	Set × Rep Target MPV (m·s^−1^) % 1RM	3 × 7 1.16 (~50% 1RM)	3 × 7 1.16 (~50% 1RM)	3 × 7 1.08 (~55% 1RM)	3 × 7 1.08 (~55% 1RM)	3 × 6 1.00 (~60% 1RM)	3 × 6 1.00 (~60% 1RM)	3 × 5 0.92 (~65% 1RM)	3 × 5 0.92 (~65% 1RM)
R_55–75_	Set × Rep Target MPV (m·s^−1^) % 1RM	3 × 7 1.08 (~55% 1RM)	3 × 7 1.08 (~55% 1RM)	3 × 6 1.00 (~60% 1RM)	3 × 6 1.00 (~60% 1RM)	3 × 6 1.00 (~60% 1RM)	3 × 5 0.92 (~65% 1RM)	3 × 5 0.92 (~65% 1RM)	3 × 5 0.92 (~65% 1RM)
R_60–70_	Set × Rep Target MPV (m·s^−1^) % 1RM	3 × 6 1.00 m·s^−1^ (~60% 1RM)	3 × 6 1.00 m·s^−1^ (~60% 1RM)	3 × 6 1.00 m·s^−1^ (~60% 1RM)	3 × 6 1.00 m·s^−1^ (~60% 1RM)	3 × 6 1.00 m·s^−1^ (~60% 1RM)	3 × 6 1.00 m·s^−1^ (~65% 1RM)	3 × 5 0.92 m·s^−1^ (~65% 1RM)	3 × 5 0.92 m·s^−1^ (~65% 1RM)
**Intensity Scheduled**		**Session 9**	**Session 10**	**Session 11**	**Session 12**	**Session 13**	**Session 14**	**Session 15**	**Session 16**
R_50–85_	Set × Rep Target MPV (m·s^−1^) % 1RM	3 × 4 0.84 (~70% 1RM)	3 × 4 0.84 (~70% 1RM)	3 × 4 0.76 (~75% 1RM)	3 × 4 0.76 (~75% 1RM)	3 × 4 0.67 (~80% 1RM)	3 × 4 0.67 (~80% 1RM)	3 × 3 0.59 (~80% 1RM)	3 × 3 0.59 (~85% 1RM)
R_55–75_	Set × Rep Target MPV (m·s^−1^) % 1RM	3 × 5 0.92 (~65% 1RM)	3 × 4 0.84 (~70% 1RM)	3 × 4 0.84 (~70% 1RM)	3 × 4 0.84 (~70% 1RM)	3 × 4 0.84 (~70% 1RM)	3 × 4 0.84 (~70% 1RM)	3 × 4 0.76 (~75% 1RM)	3 × 4 0.76 (~75% 1RM)
R_60–70_	Set × Rep Target MPV (m·s^−1^) % 1RM	3 × 5 0.92 (~65% 1RM)	3 × 5 0.92 (~65% 1RM)	3 × 5 0.92 (~65% 1RM)	3 × 5 0.84 (~70% 1RM)	3 × 4 0.84 (~70% 1RM)	3 × 4 0.84 (~70% 1RM)	3 × 4 0.84 (~70% 1RM)	3 × 4 0.84 (~70% 1RM)

*n* = 34; R_50–85_: range from 50 to 85% 1RM (*n* = 12); R_55–75_: range from 55 to 75% 1RM (*n* = 12); R_60–70_: range from 60 to 70% 1RM (*n* = 10); MPV: mean propulsive velocity; 1RM: one-repetition maximum in full squat.

**Table 3:** Descriptive characteristics of the 8-week velocity-based squat training program performed by each range of load.

Actually Performed	Fastest-MPV (m·s^−1^) [%1RM]	Average-MPV (m·s^−1^)	VL (%)	Total Reps	MRI (m·s^−1^) [%1RM]	NTF		
R_50–85_	0.88 ± 0.01 ^Ψ^ ♣ [~67.5% 1RM]	0.96 ± 0.02	21.4 ± 6.1	238.8 ± 1.6 ^Ψ^	0.93 ± 0.01 [~65% 1RM]	1.2 ± 1.6 ^Ψ^		
R_55–75_	0.91 ± 0.01 ^Ψ^ [~65% 1RM]	0.96 ± 0.02	21.1 ± 5.4	239.6 ± 0.9	0.93 ± 0.01 [~65% 1RM]	0.9 ± 0.3		
R_60–70_	0.92 ± 0.01 [~65% 1RM]	0.96 ± 0.02	18.1 ± 6.1	240.0 ± 0.0	0.93 ± 0.01 [~65% 1RM]	0.0 ± 0.0		
**Actually Performed**	**Rep per set with 50% 1RM**	**Rep per set with 55% 1RM**	**Rep per set with 60% 1RM**	**Rep per set with 65% 1RM**	**Rep per set with 70% 1RM**	**Rep per set with 75% 1RM**	**Rep per set with 80% 1RM**	**Rep per set with 85% 1RM**
R_50–85_	7.0 ± 0.0	7.0 ± 0.0	6.0 ± 0.0	5.0 ± 0.0	4.0 ± 0.0	4.0 ± 0.1	3.8 ± 0.4	3.0 ± 0.1
R_55–75_		7.0 ± 0.0	6.0 ± 0.0	5.0 ± 0.0	4.0 ± 0.0	3.9 ± 0.1		
R_60–70_			6.0 ± 0.0	5.0 ± 0.0	4.0 ± 0.0			

Data are mean ± SD *n* = 34. R_50–85_: range from 50 to 85% 1RM (*n* = 12). R_55–75_: range from 55 to 75% 1RM (*n* = 12). R_60–70_: range from 60 to 70% 1RM (*n* = 10). Fastest-MPV: average of the fastest mean propulsive velocity (MPV) measured in each session, which corresponds with the intensity (%1RM) scheduled. Average-MPV: average MPV attained during the entire training program. VL: velocity loss in the set calculated as a percent loss in mean velocity from the fastest to the last repetition of each set. Total Reps: total number of repetitions performed during the training program. MRI: mean relative intensity calculated as the weighted average of the repetitions performed with each %1RM (this value is an indicator of the average loading magnitude, %1RM, achieved during the training program). NTF: number of times to failure. Rep per set with a given %1RM: number of repetitions performed in each set with each relative load (50–85%1RM). Significant differences compared to the R_60–70_: ^Ψ^ (*p* < 0.05). Significant differences compared to the R_55–75_: ♣ (*p* < 0.05).

**Table 4:** Changes in selected performance variables from pre- to post-training for each range of load.

	R_50–85_ (n = 12)			R_55–75_ (n = 12)			R_60–70_ (n = 10)				
	Pre	Post	Δ (%)	Pre	Post	Δ (%)	Pre	Post	Δ (%)	*p*-Value Time Effect	*p*-Value Group × Time
1RM (kg)	90.6 ± 19.4	108.3 ± 16.8 ***	19.6	90.4 ± 22.1	102.8 ± 20.2 ***	13.6	84.0 ± 13.8	97.1 ± 15.5 ***	15.6	<0.001	0.23
AV (m·s^−1^)	0.95 ± 0.12	1.15 ± 0.08 *** ^Ψ^	21.1	0.98 ± 0.09	1.09 ± 0.07 **	11.2	0.94 ± 0.05	1.05 ± 0.10 **	11.7	<0.001	<0.004
AV > 1 (m·s^−1^)	1.25 ± 0.09	1.40 ± 0.09 **	12.0	1.30 ± 0.08	1.35 ± 0.06 *	3.8	1.26 ± 0.07	1.35 ± 0.10 *	7.1	<0.001	0.33
AV < 1 (m·s^−1^)	0.68 ± 0.03	0.91 ± 0.12 ***	34.0	0.67 ± 0.05	0.83 ± 0.07 ***	23.9	0.69 ± 0.03	0.85 ± 0.08 ***	23.1	<0.001	0.41
CMJ (cm)	36.2 ± 5.3	39.0 ± 4.9 ***	7.7	34.1 ± 4.6	36.4 ± 4.3 ***	6.8	32.2 ± 3.5	35.1 ± 5.2 ***	8.9	<0.001	0.85
T_10_ (s)	1.79 ± 0.09	1.79 ± 0.07	0.0	1.83 ± 0.12	1.81 ± 0.09	−1.1	1.84 ± 0.08	1.80 ± 0.07	−2.2	<0.05	0.36
T_20_ (s)	3.09 ± 0.14	3.09 ± 0.12	0.0	3.16 ± 0.18	3.11 ± 0.14	−1.6	3.18 ± 0.12	3.10 ± 0.11 **	−2.5	<0.01	0.12
T10–20 (s)	1.30 ± 0.07	1.29 ± 0.05	−0.8	1.32 ± 0.07	1.30 ± 0.06 **	−1.5	1.34 ± 0.06	1.30 ± 0.05 ***	−3.0	<0.002	0.13

Data are mean ± SD, *n* = 34. R_50–85_: range from 50 to 85% 1RM (*n* = 12). R_55–75_: range from 55 to 75% 1RM (*n* = 12). R_60–70_: range from 60 to 70% 1RM (*n* = 10). 1RM: one-repetition maximum in full squat exercise. MPV: mean propulsive velocity. AV: average MPV attained against absolute loads common to pre- and post-training in the progressive loading test. AV > 1: average MPV attained against absolute loads moved faster than 1 m·s^−1^ at pre-training. AV < 1: average MPV attained against absolute loads moved slower than 1 m·s^−1^ at pre-training. CMJ: countermovement jump height. T10: 10 m sprint time. T20: 20 m sprint time. T10–20: 10 to 20 m sprint time. Intra-group significant differences from pre to post: * *p* < 0.05, ** *p* < 0.01, *** *p* ˂ 0.001. Significant differences compared to the R_60–70_: ^Ψ^ (*p* < 0.05).

## Data Availability

The data showed in this study are available on request from the corresponding author. The data are not publicly available due to containing information that could compromise the privacy of research participants.
